# A non-invasive diagnostic tool for the assessment of optimal positive-end expiratory pressure (PEEP_OPT_) in infants receiving prolonged invasive ventilation

**DOI:** 10.1038/s41372-026-02579-z

**Published:** 2026-02-16

**Authors:** Nada Darwish, Ann Donnelly, Jennifer Erkinger, Shaoyi Zhang, Dajiang Liu, Jeffrey R. Kaiser, Roopa Siddaiah

**Affiliations:** 1 Division of Neonatal-Perinatal Medicine, Penn State Health Children’s Hospital, Hershey, PA USA; 2 Department of Respiratory Care, Penn State Health Children’s Hospital, Hershey, PA USA; 3 Department of Public Health Sciences, Penn State College of Medicine, Hershey, PA USA; 4 Department of Pediatrics, Penn State College of Medicine, Hershey, PA USA; 5 Division of Pediatric Pulmonology, Arkansas Children’s Hospital, Hershey, PA USA

**Keywords:** Paediatrics, Respiration, Respiratory tract diseases, Therapeutics, Diagnosis

## Abstract

**Objective:**

Establish the feasibility of optimizing PEEP (PEEP_OPT_) using ventilator software in infants receiving prolonged invasive mechanical ventilation.

**Methods:**

A retrospective review of infants who underwent PEEP_OPT_ studies. Paired-t test was used to compare dynamic lung compliance (Cdyn), tidal volume (VT), and oxygen saturation (SpO_2_) before and after PEEP_OPT_ studies.

**Results:**

Fifteen infants with bronchopulmonary dysplasia and/or receiving prolonged invasive mechanical ventilation were included with a median gestational age of 24 4/7 weeks and postmenstrual age of 40 1/7 weeks at the time of the PEEP_OPT_ study. PEEP_OPT_ was different from clinically set PEEP (PEEP_SET_) in 14/15 infants; PEEP_OPT_ was higher in 27% and lower in 67% of infants (–2 to +5cmH_2_O). Cdyn improved for most infants by 0.23 (0.01–0.60) mL/H_2_O (*p* < 0.001). VT increased for most infants by 1.5 (–0.5 to 3.4) mL/kg (*p* < 0.001), and SpO_2_ increased by 2% (–1 to 12%).

**Conclusion:**

Assessment of PEEP_OPT_ using our standardized protocol is feasible, non-invasive, and provides a low-cost bedside tool. PEEP_OPT_ differed from PEEP_SET_ in most subjects, highlighting the importance of evaluating PEEP_OPT_.

## Introduction

Premature infants receiving prolonged invasive mechanical ventilation are at risk for developing bronchopulmonary dysplasia (BPD). BPD is a heterogeneous chronic lung disease that is often challenging to manage due to the difficulty of establishing the optimal level of positive end-expiratory pressure (PEEP) needed to maintain airway patency and adequate gas exchange during mechanical ventilation.

Determining optimal PEEP (PEEP_OPT_) is a vital aspect of managing ventilated infants with BPD. Too little PEEP can lead to airway obstruction and collapse, atelectasis, subsequent atelectotrauma, ventilation and oxygenation deficits, and worsening lung damage [1]. It may also hinder the ability to trigger breaths from flow-cycled ventilators, since infants with the obstructive-phenotype of BPD often exhibit dynamic hyperinflation and subsequently high intrinsic PEEP (i.e., auto-PEEP) [2]. This may result in patient-ventilator asynchrony, leading to further ventilation-perfusion mismatch, increased agitation, and need for sedative agents. In addition, premature infants receiving prolonged mechanical ventilation often develop tracheobronchomalacia, which has been reported to occur in 10-86% of infants undergoing bronchoscopy [3–7]. Tracheobronchomalacia compounds the ventilatory management of premature infants with BPD [8], and PEEP_OPT_ is then needed to sustain airway patency and alveolar recruitment, once critical opening pressure is overcome, allowing for exhalation [9].

Excess PEEP, on the other hand, can cause lung hyperinflation and alveolar overdistention which can lead to air trapping, inability to ventilate and oxygenate, and exacerbation of lung injury [1, 10]. Lung hyperinflation and subsequent increased intrathoracic pressure impedes venous return to the heart and may lower cardiac output leading to systemic hypotension [1, 10]. The high intrathoracic pressure also results in higher pulmonary vascular resistance (PVR) and increased right ventricular afterload. The PEEP_OPT_ is key in improving the overall PVR and overcoming airway-malacia, while preventing alveolar collapse and resultant hypoxic vasoconstriction from too low PEEP, as well as preventing lung hyperinflation and reduced venous return with too high PEEP. Thus, determining PEEP_OPT_ is essential in the management of premature infants receiving prolonged mechanical ventilation.

The Servo-U ventilator (Getinge, Gothenburg, Sweden) has an algorithm that can be leveraged to evaluate PEEP_OPT_ in mechanically ventilated infants using a simple test protocol. This allows for the rapid determination of PEEP_OPT_ at the bedside in infants using only the ventilator as an assessment tool, therefore avoiding the risks associated with other methods of determining PEEP_OPT_ that include radiation and being transferred to the radiology suite or operating room. We developed a protocol using the Servo-U ventilator to evaluate PEEP_OPT_ in infants receiving prolonged invasive mechanical ventilation in our neonatal intensive care unit (NICU). The goal of our study was to assess the impact of the PEEP_OPT_ protocol on immediate clinical ventilatory outcomes in premature infants receiving prolonged invasive mechanical ventilation.

## Methods

This is a retrospective review of all infants who underwent PEEP_OPT_ studies per our protocol in our level IV NICU from January 2022 to January 2025. This study was approved by the Penn State Health Institutional Review Board. Indications for undergoing a PEEP_OPT_ study were determined by neonatologists based on clinical presentations such as respiratory instability, atelectasis or hyperinflation on chest x-ray, and increased oxygen needs.

### Patient population

Eligible infants were identified by neonatologists in the Penn State Health Children’s Hospital level IV NICU in Hershey, Pennsylvania. Infants who received PEEP_OPT_ studies were premature infants born at ≤32 weeks gestation who were receiving prolonged invasive mechanical ventilation (defined as >7 days) with challenges in achieving adequate oxygenation and ventilation, infants with Grade 3 BPD [8], and infants who newly underwent tracheostomy tube placement during the first 5 postoperative days while receiving sedation and paralysis per our NICU protocol before their first tracheostomy tube change. All infants recruited were receiving conventional mechanical ventilation using the Servo-U ventilator. Infants with severe congenital anomalies and chromosomal abnormalities were excluded.

### Optimal PEEP protocol

To ensure accuracy of this assessment tool, infants were sedated and received pharmacologic paralysis during the study to prevent spontaneous breathing above the set ventilator rate. In those receiving a continuous sedative infusion, a bolus equivalent of an hourly dose was given. In infants receiving scheduled or PRN doses of sedation/analgesic agents, these drugs were given at the beginning of the study. Other infants received single doses of an analgesic (fentanyl or morphine) and an anxiolytic (midazolam, lorazepam, or dexmedetomidine) agent. All infants received vecuronium for pharmacologic paralysis at the beginning of the study.

Prior to the study, infants receiving PEEP_OPT_ studies were transitioned to pressure control ventilation (synchronized intermittent mandatory ventilation-pressure control with pressure support [SIMV (PC) + PS]), if not already receiving it, for the duration of the study. When transitioning infants who were receiving volume-targeted ventilation to pressure control ventilation, the average peak inspiratory pressure (PIP) was used. This enabled the team to determine the impact of changing PEEP on VT.

The study was carried out at the bedside by trained Respiratory Therapists using the following protocol:

1. The Open Lung Tool Trends screen on the Servo-U ventilator was used. Baseline information was collected from the medical record, cardiorespiratory monitor, and ventilator. Starting at the patient’s currently PEEP_SET_, dynamic compliance (Cdyn), exhaled tidal volumes (VTe), oxygen saturation (SpO_2_), and non-invasive vital signs were recorded.
2. PEEP was increased by 1 cm H_2_O above the currently PEEP_SET_, and PIP was similarly increased to maintain deltaP.
3. The parameters listed above were recorded after stabilization over 3–5 min. A manual blood pressure was obtained and recorded at the end of each time interval.

This time interval was chosen based on a study of adults with acute respiratory distress syndrome (ARDS) where PaO_2_ and lung compliance equilibrated within 5 min of a PEEP change [11]. There are no similar studies on the time to achieve equilibrium following PEEP changes in infants, but it is likely similar if not shorter in duration. Importantly, we hoped to avoid redosing sedation and paralytics which would have been necessary with longer studies.

4. Step 2 was repeated as PEEP was increased in increments of 1 cm H_2_O every 3–5 min, observing and trending the parameters listed above until either of the following occurred:

- A change in vital signs from baseline: increased or decreased heart rate (by 20 bpm), decrease in oxygen saturations (by >10%), or a decrease in systolic blood pressure (by >20 mmHg).

5. If there was no improvement or worsening of Cdyn or SpO_2_ when PEEP was increased above the PEEP_SET_, PEEP (and PIP) was then decreased in increments of 1 cm H_2_O every 3–5 min (from PEEP_SET_), and respiratory and clinical parameters were similarly recorded as above. If Cdyn improved by increasing PEEP, then step 5 was not necessarily completed.
6. The PEEP level where Cdyn was most optimal, together with appropriate vital signs, was considered the PEEP_OPT_.
7. All data were recorded in the infant’s electronic medical record.

### Statistical analysis

Statistical analysis was performed using paired t-tests to compare respiratory parameters between clinically PEEP_SET_ and PEEP_OPT_ values. The primary outcome measure was Cdyn, and secondary outcome measures were VT and SpO_2_. Clinical parameters such as heart rate, respiratory rate, and blood pressure were measured to ensure safety and tolerance. Changes in these parameters between PEEP_SET_ and PEEP_OPT_ settings were assessed, and a *p* value < 0.05 was considered statistically significant.

To assess the magnitude of clinical improvement, the mean difference in Cdyn, VT, and SpO_2_ between the PEEP_SET_ and PEEP_OPT_ was calculated. Statistical analyses were conducted using SPSS, V29 (IBM, Armonk, NY).

## Results

A total of 15 infants were included in this retrospective review. Demographic characteristics are shown in Table 1. The median gestational age at birth was 24 4/7 (range 22 0/7–29 6/7) weeks gestation with a median postmenstrual age of 40 1/7 (range 30 3/7–49 5/7) weeks at the time of the study. Most infants (87%) met criteria for the diagnosis of grade 3 BPD [8] at the time of the study; two infants were <36 weeks’ postmenstrual age. The mean number of failed extubation events prior to the PEEP_OPT_ study was two per infant. Four infants had direct laryngobronchoscopy evaluation prior to the study (including prior to tracheostomy placement) which revealed airway granulomas. Three infants (20%) had a tracheostomy tube, eight infants (53%) had a cuffed endotracheal tube, and four infants (27%) had an uncuffed endotracheal tube at the time of the study. In infants with tracheostomy or cuffed endotracheal tubes, the cuff was inflated in 3/11 infants (27%) with a mean of 1.4 mL (0–2 mL) of water. Measured cuff pressures were within manufacturer recommendations. All infants had minimal to no leak except one infant with a 30% leak around a 4.0 mm endotracheal tube. Infants were receiving ventilation with low rate (22 ± 7 bpm), high VT, and long inspiratory time (0.6 ± 0.1 s).

**Table 1:** Baseline characteristics of the study population.

Demographics	Value
GA (weeks), median (range)	24 4/7 (22 0/7–29 6/7)
PMA at the time of study (weeks), median (range)	40 1/7 (30 3/7–49 5/7)
Female, n (%)	5 (33%)
Weight at the time of study (g), mean (SD)	3452 ± 1544
BPD Grade III, n (%)	13 (87%)*
Failed extubation per infant, mean (SD)	2 ± 1
Airway granuloma, n (%)	4 (27%)
Artificial airway type at time of study	
Tracheostomy tube, n (%)	3 (20%)
Endotracheal tube, cuffed; n (%)	8 (53%)
Endotracheal tube, uncuffed; n (%)	4 (27%)
Ventilation mode	
SIMV (PC) + PS, n (%)	10 (67%)
SIMV (PRVC) + PS, n (%)	5 (33%)
Ventilatory settings	
Pressure control (cmH_2_O), mean (SD)	26 ± 7
Respiratory rate (bpm), mean (SD)	22 ± 1
Inspiratory time (s), mean (SD)	0.6 ± 0.1

^*^Remaining infants <36 weeks PMA.

*SD* standard deviation, *GA* gestational age, *PMA* post-menstrual age, *BPD* bronchopulmonary dysplasia, *SIMV* synchronized intermittent mandatory ventilation, *PC* pressure control, *PRVC* pressure-regulated volume control.

All infants received fentanyl for sedation and vecuronium for pharmacologic paralysis prior to the study (Table 2). Additionally, infants either received midazolam (87%), dexmedetomidine (20%), or lorazepam (13%) as an adjunct agent. Most infants received two sedation agents, and one infant received four agents (two were scheduled medications, and two were given in addition due to inadequate sedation prior to the study).

**Table 2:** Sedation and paralysis agents used prior to the study.

Sedation and paralysis	*N* (%)
Sedation Agents, type	
Fentanyl	15 (100%)
Midazolam	13 (87%)
Dexmedetomidine	3 (20%)
Lorazepam	2 (13%)
Morphine	1 (7%)
Sedation Agents, number	
Two agents	12 (80%)
Three agents	2 (13%)
Four agents	1 (7%)
Paralysis, Vecuronium	15 (100%)

**Fig. 1:**
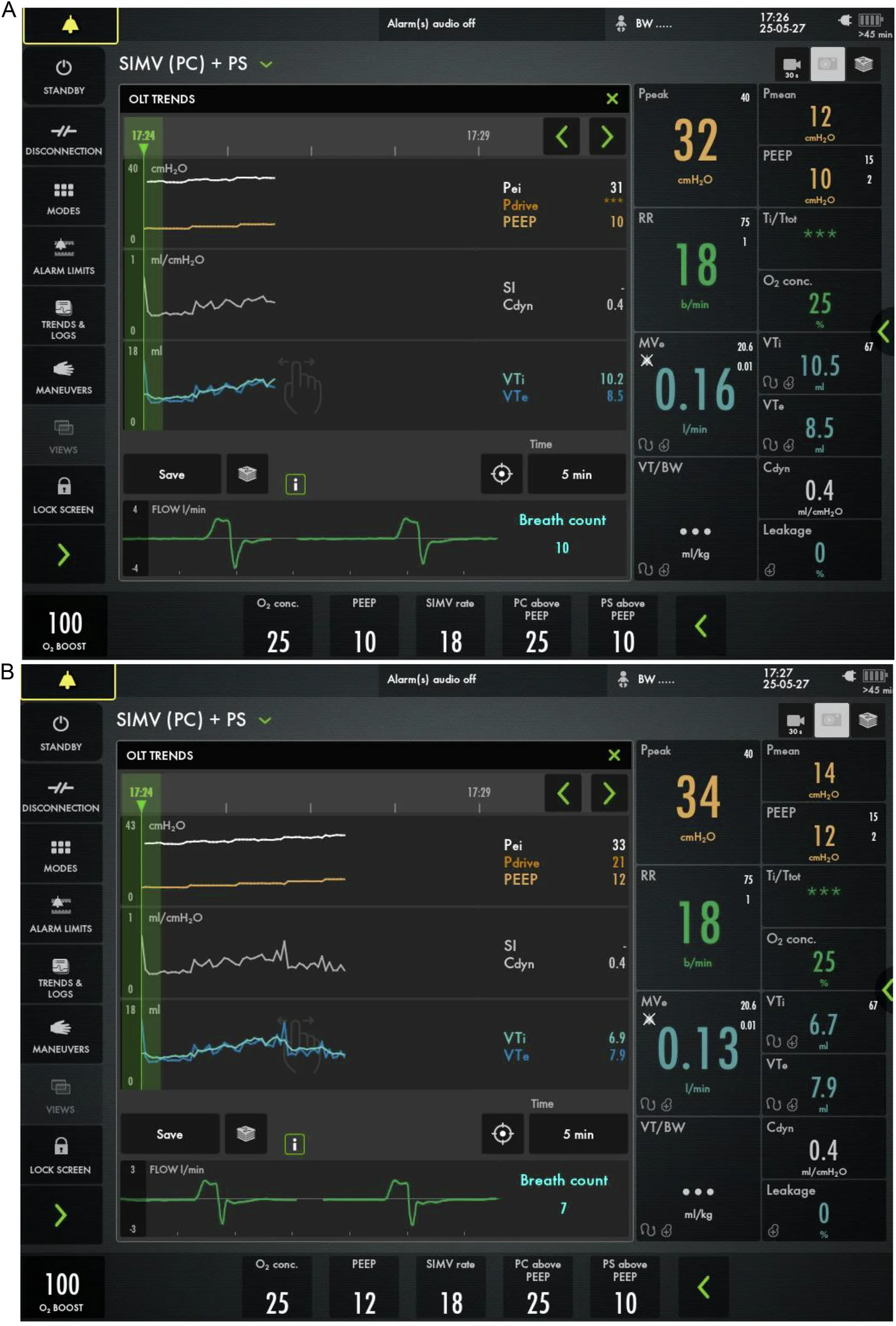
Simulated PEEP_OPT_ study using the Servo-U ventilator. In this study, the PEEP_OPT_ was 10 cmH_2_O as determined by the Cdyn being at its highest. At this PEEP, VTe was also found to be at its highest. **A** PEEP is gradually increased from 8 to 10 cmH_2_O, with subsequent increase in Cdyn. **B** As PEEP is increased gradually beyond 10 cmH_2_O to 12 cmH_2_O, Cdyn and VTe decrease.

Figure 1 demonstrates the ventilator’s Open Lung Tool screen during a simulated PEEP_OPT_ study using a neonatal test lung. For this study, PEEP_SET_ was 8 cmH_2_O. PEEP was increased sequentially from the PEEP_SET_ to 12 cmH_2_O, and PIP was similarly increased with PEEP changes to maintain the same deltaP. Cdyn was noted with every increase, as well as VTe. Cdyn was highest at a PEEP of 10 cmH_2_O, hence, this was determined to be PEEP_OPT_ in this representative study. At this PEEP, VTe was the highest. Following subsequent increases in PEEP to 11 and 12 cmH_2_O, Cdyn declined, due to hyperinflation, further confirming the PEEP_OPT_ to be 10 cmH_2_O.

In our study cohort, PEEP_OPT_ was different from the clinically PEEP_SET_ in all but one infant (Table 3), with the change in PEEP ranging from –2 to +5 cmH_2_O. The PEEP_OPT_ was lower in 10 of the infants (67%), and higher in four of the infants (27%) from the PEEP_SET_. Of the 14 infants where PEEP_OPT_ was different than PEEP_SET_, Cdyn improved in all infants at PEEP_OPT_ compared to the PEEP_SET_ by an average of 0.23 (0.01–0.60) mL/H_2_O (*p* < 0.001) as shown in Table 4. The VT achieved was significantly higher when using PEEP_OPT_ rather than PEEP_SET_ (*p* < 0.001) with an average increase of 5.3 (–1 to 14) mL or 1.5 (–0.5 to 3.4) mL/kg. SpO_2_ similarly increased in most infants by an average of 2% (–1 to 12%) using PEEP_OPT_, however, this did not reach statistical significance (*p* > 0.05).

**Table 3:** Change in PEEP following the optimal PEEP maneuver.

Baseline and change in PEEP	Value
Set PEEP (mmHg), mean ∓ SD	10 ± 2
Change in PEEP	
Increased, n (%)	4 (27%)
Decreased, n (%)	10 (67%)
No Change, n (%)	1 (7%)

*SD* standard deviation, *PEEP* positive-end expiratory pressure, *FiO2* fraction of inspired oxygen.

**Table 4:** Clinical parameters prior to and following the optimal PEEP study.

Subject	1	2	3	4	5	6	7	8	9	10	11	12	13	14	15	P-value
PEEP (mmH2O)																
Set	11	8	8	11	9	12	12	10	10	9	14	8	9	10	9	-
Optimal	10	6	13	9	8	14	10	11	10	7	13	10	8	9	7	
Change	–1	–2	+5	–2	–1	+2	–2	+1	0	–2	–1	+2	–1	-1	-2	
Cdyn (mL/H2O)																
Set	3.0	2.9	1.2	2.1	1.4	0.83	3	0.8	0.9	0.9	2.9	1.1	0.5	1.4	0.9	0.000*
Optimal	3.4	3.2	1.8	2.6	1.6	0.84	3.1	1.3	0.9	1.1	3	1.3	0.6	1.6	1.0	
Change	0.4	0.3	0.6	0.5	0.2	0.01	0.1	0.5	0	0.2	0.1	0.2	0.1	0.2	0.1	
VT, mL (mL/kg)																
Set	63 (12.4)	40 (9.1)	25 (5.8)	59 (14.5)	48 (13.3)	23 (8.5)	62 (9.6)	25 (7.9)	18.8 (12.7)	19.3 (14.5)	56 (11.6)	31 (14.0)	11.4 (6.6)	37 (8.0)	17.1 (9.7)	0.000*(0.000*)
Optimal	71 (14.0)	45 (10.2)	38 (8.8)	73 (18.0)	56 (15.5)	27 (10.0)	62 (9.6)	33 (10.5)	18.8 (12.7)	22 (16.5)	59 (12.2)	30 (13.5)	16.1 (9.3)	46 (9.9)	17.7 (10.1)	
Change	8 (1.6)	5 (1.1)	13 (3.0)	14 (3.4)	8 (2.2)	4 (1.5)	0 (0.0)	8 (2.5)	0 (0.0)	2.7 (2.0)	3.0 (0.6)	-1.0 (-0.5)	4.7 (2.7)	9.0 (1.9)	0.6 (0.3)	
SpO2																
Set	98	97	87	93	96	95	93	94	99	N/A	100	99	100	90	95	0.05
Optimal	98	99	99	96	97	97	93	98	99		100	100	100	92	94	
Change	0	2	12	3	1	2	0	4.0	0		0	1	0	2	-1	
FiO2	46	24	40	55	31	65	25	60	68	44	40	75	50	36	30	-

*PEEP* positive-end expiratory pressure, *Cdyn* dynamic compliance, *VT* tidal volume, *SpO*_2_ oxygen saturation by pulse oximeter, *FiO*_2_ fraction of inspired oxygen.

*p < 0.05.

Blood pressure was trended as a marker for potential cardiovascular compromise with changing PEEP and was found to be similar or slightly increased with increasing PEEP.

## Discussion

The definition of PEEP_OPT_ is controversial, specifically as it pertains to lung compliance and oxygenation, where the PEEP at which lung compliance is best may not be the same PEEP at which oxygenation is best. One study found that oxygenation-based PEEP_OPT_ tends to be higher than compliance-based PEEP_OPT_ [12]. This is likely because compliance-based PEEP_OPT_ reflects the PEEP_OPT_ at which the alveoli are recruited but not overdistended. It reflects optimization of respiratory mechanics and the balance between shunting and dead space, accounting for different areas of the lung with different time constants. That is, whereas oxygenation-based PEEP_OPT_ optimizes lung recruitment and reduces intrapulmonary shunting, it does not necessarily decrease mechanical stress. Oxygenation-based PEEP_OPT_ may improve oxygenation at the expense of worsening alveolar overdistention, hence worsening CO_2_ clearance and higher risk of ventilator-induced lung injury [12]. This is especially relevant in heterogeneous lung disease such as BPD, where compliance-based PEEP_OPT_ may be more protective. In our study, we consider PEEP_OPT_ as the level of PEEP that effectively maintains alveolar recruitment at the end of expiration while minimizing alveolar overdistention and air trapping. Thus, we chose Cdyn as our primary endpoint.

There is limited guidance from clinical research studies on selecting the PEEP_OPT_, leading to substantial variability in PEEP levels used among different centers and providers [10, 13]. Since PEEP_OPT_ can be influenced by a variety of factors in each infant, an individualized approach is necessary. In our preliminary feasibility study, we present a simple non-invasive bedside tool that can be used to determine PEEP_OPT_ in infants receiving invasive mechanical ventilation. While we used the Servo-U ventilator, other modern neonatal ventilators have similar capabilities. Using our protocol, we were able to determine PEEP_OPT_ and demonstrate correlating improvement in Cdyn and VT. Achieving PEEP_OPT_ allows for sustained alveolar recruitment once the critical opening pressure is overcome, prevention of alveolar collapse, improved Cdyn, and subsequently greater VT.

There is a well-established correlation between lung volume and PVR. As lung volume increases, the distending alveolar units compress alveolar vessels increasing the vascular resistance whereas extra-alveolar vessels dilate, achieving the lowest PVR at the pulmonary functional residual capacity [14]. Too low PEEP will result in higher overall PVR due to higher resistance in extra-alveolar vessels [15]. Likewise, too much PEEP will result in increasing PVR due to resistance from compressed alveolar vessels [15]. PEEP_OPT_, on the other hand, results in decreased PVR due to improved lung recruitment [15].

To date, determining PEEP_OPT_ at the bedside, without transferring infants from the controlled environment of the NICU and not using ionizing radiation or additional experimental technology has been an elusive goal. There is a great need for determining PEEP_OPT_ using a non-invasive, cheap, and practical method that can provide a better reflection of the dynamic PEEP requirement of individual infants during varying clinical states. In this paper, we describe a standardized non-invasive method of estimating PEEP_OPT_ at the bedside that can be easily repeated as clinical status changes. Assessment of PEEP_OPT_, by titrating PEEP levels to find the optimal Cdyn, while maintaining adequate vital signs, is therefore an important maneuver to manage the long-term ventilation of individuals with severe BPD and tracheobronchomalacia.

The diagnosis of tracheobronchomalacia is typically made by direct visualization during laryngobronchoscopy in the operating room. This invasive procedure, however, is often subjective with low interrater reliability yielding little usable quantitative data [8, 16]. Further, it is typically a static study, not providing dynamic airway information by varying levels of PEEP. Laryngobronchoscopy also carries significant risk in infants with BPD who have high oxygen requirements, high mean airway pressure, and sometimes pulmonary hypertension, by transporting them from the controlled NICU environment to the operating room. Chest CT has similarly been used to assess the severity of tracheobronchomalacia and determine the adequacy of the provided PEEP in mechanically ventilated infants [7, 17, 18]. While this invasive approach allows for the evaluation of PEEP_OPT_, it involves radiation exposure, requires the development of specialized protocols for scanning and interpretation, is costly, not easily repeated when clinical status changes, and requires transfer to the radiology suite with several accompanying NICU providers.

In medical practice, intensivists rely on clinical parameters such as oxygen requirement, intermittent blood gases, and appearance on chest radiographs to determine PEEP level [19]. But we have shown in this study that clinical determination of PEEP_OPT_ is an imperfect science, and that PEEP_SET_ has been both lower and higher than the PEEP_OPT_ determined by our method in most of our patients. Our findings are consistent with previous studies which used other methods to determine PEEP_OPT_ [7, 17, 18]. Clinicians often feel inclined to increase PEEP to treat signs of respiratory distress and air hunger, or to achieve better lung recruitment [19]. In fact, in our cohort, the PEEP_OPT_ was more often lower than the PEEP_SET_. This further highlights the need for a standardized easily replicable assessment of PEEP_OPT_ to prevent exposure of lungs to alveolar overdistention and air trapping.

In some NICUs, efforts to elucidate PEEP_OPT_ may include changing PEEP and examining subsequent changes in Cdyn and VT (at a given deltaP), as well as pressure-volume loops. However, the approaches for PEEP titration are not standardized. More recently, lung ultrasound (LUS) is playing an increasingly prominent role in guiding respiratory therapies. Like our protocol, lung ultrasound is another non-invasive tool that provides real-time detection of lung aeration and has been used to guide PEEP titration in pediatrics studies [20–22]. However, LUS is operator-dependent and requires training and experience in image acquisition and interpretation [22, 23]. While LUS may provide quantification data of lung volumes, it does not measure compliance, does not distinguish overdistension from normal aeration, and may miss consolidation and BPD changes in deeper areas of the lung that does not extend to the pleura [20, 22, 23]. Electrical impedance tomography (EIT) is another increasingly popular method that allows radiation-free imaging of a cross-section of the lungs to examine lung volumes in the neonatal population [24, 25] and has been used in PEEP titration [26]. However, EIT suffers from similar limitations to LUS, with need for training and experience as well as cost and availability of equipment limiting its use [24]. In addition, EIT detects changes in impedance (i.e., air content) but not compliance, and may not distinguish recruitment and overdistention [25]. Furthermore, none of the existing methods have been standardized in neonates [25].

The main limitation of our approach, as well as the other approaches, is that it evaluates PEEP_OPT_ in a sedated and paralyzed state and does not directly inform PEEP requirement for awake infants. Using this tool arguably allows clinicians to determine the minimal PEEP required when infants are in a calm, non-disturbed state. Our protocol, different from the other techniques, additionally provides a dynamic evaluation of lung compliance that considers the change in lung mechanics with changing PEEP. It is also important to recognize that once PEEP_OPT_ is established, it is likely to change as the lungs of chronically ventilated infants grow and heal, or during clinical decompensation (e.g., infection). Our protocol allows for repeat determination of PEEP_OPT_ at the bedside in a standardized, relatively non-invasive, easy, practical and cheap manner, without the side effects of other methods, as infants continue receiving chronic mechanical ventilation. Lastly, we were able to demonstrate short-term improvement in lung compliance and VT with PEEP optimization using our protocol. However, whether this effect persists beyond the study period remains unknown due to the short time interval between each PEEP change which was limited to avoid redosing sedation and paralytics.

We suggest that our preliminary results be used to guide future larger studies to evaluate how our proposed standardized protocol compares to results obtained from other diagnostic tools. Further studies are also needed to elucidate how often PEEP_OPT_ studies need to be repeated to capture the change in PEEP_OPT_ in periods of clinical illness and lung healing. Future research may additionally examine the impact of using this protocol on short-term outcomes, such as ventilation and oxygen requirements, and long-term outcomes such as days of ventilation, length of stay, sedation requirements, need for tracheostomy placement, and neurodevelopmental outcomes.

## Conclusion

Assessment of PEEP_OPT_ using the ventilator is feasible, safe, easily repeatable, and provides a non-invasive, non-costly, bedside tool to aid PEEP optimization in infants with BPD and/or tracheobronchomalacia. PEEP_OPT_ was found to be different in fourteen of fifteen infants, highlighting how clinical determination of PEEP is often flawed, and underscoring the importance of finding PEEP_OPT_.

## Data Availability

Most data generated or analyzed during this study are included in this published article. All data not included are available from the corresponding author upon reasonable request.
